# Uptake and impact of vaccinating primary school children against influenza: Experiences in the fourth season of the live attenuated influenza vaccination programme, England, 2016/2017

**DOI:** 10.1111/irv.12898

**Published:** 2021-08-17

**Authors:** Mary A. Sinnathamby, Fiona Warburton, Nick Andrews, Nicola L. Boddington, Hongxin Zhao, Joanna Ellis, Elise Tessier, Matthew Donati, Alex J. Elliot, Helen E. Hughes, Rachel Byford, Gillian E. Smith, Manasa Tripathy, Simon de Lusignan, Maria Zambon, Richard G. Pebody

**Affiliations:** ^1^ Immunisation and Countermeasures, National Infection Service Public Health England (PHE) London UK; ^2^ Statistics and Modelling Department, National Infection Service Public Health England (PHE) London UK; ^3^ Virus Reference Department, National Infection Service Public Health England (PHE) London UK; ^4^ National Infection Service Laboratories, National Infection Service Public Health England (PHE) Bristol UK; ^5^ Real‐Time Syndromic Surveillance Team, National Infection Service Public Health England (PHE) Birmingham UK; ^6^ Nuffield Department of Primary Care Health Sciences University of Oxford Oxford UK; ^7^ Research and Surveillance Centre (RSC) Royal College of General Practitioners (RCGP) London UK

**Keywords:** influenza vaccination, live attenuated influenza vaccine, vaccine impact, vaccine uptake

## Abstract

**Background:**

In the 2016/2017 influenza season, England was in its fourth season of the roll‐out of a live‐attenuated influenza vaccine (LAIV) targeted at healthy children aged two to less than 17 years. For the first time, all healthy children aged 2 to 8 years were offered LAIV at national level in 2016/2017. Since the commencement of the programme in 2013/2014, a series of geographically discrete pilot areas have been in place where quadrivalent LAIV was also offered to all school age children. In 2016/2017, these were children aged 8 to 11 years, other than those targeted by the national programme.

**Methods:**

We evaluated the overall and indirect impact of vaccinating primary school age children, on the population of England, by measuring vaccine uptake levels and comparing cumulative disease incidence through various influenza surveillance schemes, in targeted and non‐targeted age groups in pilot and non‐pilot areas in 2016/2017.

**Results:**

Our findings indicate that cumulative primary care influenza‐like consultations, primary and secondary care swab positivity, influenza confirmed hospitalisations and emergency department attendances in pilot areas were overall lower than those observed in non‐pilot areas; however, significant differences were not always observed in both targeted and non‐targeted age groups. Excess mortality was higher in pilot areas compared with non‐pilot areas.

**Conclusions:**

These results are similar to earlier seasons of the programme indicating the importance and continuing support of vaccinating all primary school children with LAIV to reduce influenza related illness across the population, although further work is needed to understand the differences in excess mortality.

## 1 INTRODUCTION

The United Kingdom (UK) commenced the incremental introduction of a new childhood influenza vaccine programme in the 2013/2014 influenza season, with the ultimate objective of offering the newly licensed intra‐nasally administered live attenuated influenza vaccine (LAIV) to all healthy children aged 2 to less than 17 years. This annual programme aimed to protect children against infection and indirectly protect those in other age groups, in particular those who are at higher risk of severe disease.^1^, ^2^ The Joint Committee on Vaccination and Immunisation (JCVI) made the recommendation to implement the programme based on a range of evidence, including an analysis of the burden of influenza by age group^3^ and mathematical modelling predicting the reduction of the burden of influenza and its future benefits, alongside an economic evaluation.^4^

Epidemiological and mathematical modelling studies have supported the concept that vaccinating children can play a key role in reducing the burden of influenza across all ages; however, there has only been limited evidence on the impact of such programmes at national level in other countries to date.^5^, ^6^, ^7^, ^8^, ^9^, ^10^, ^11^

The 2016/2017 influenza season was the fourth season of introducing the LAIV vaccine in the UK. England extended the childhood vaccine programme nationally to include all healthy children aged 2 to 4 years as well as to children of school years 1–3 (aged 5 to 8 years).^12^ In addition to the national programme, five geographically discrete pilot areas in England vaccinated the remaining healthy children of primary school age in school years 4 to 6 (aged 8 to 11 years) in 2016/2017.

The 2016/2017 season was dominated by the circulation of influenza A(H3N2), the impact of which predominately affects older persons. There were increased care home outbreaks, hospital admissions and excess mortality in this age group.^13^ Vaccine effectiveness (VE) estimates for the 2016/2017 season in children were encouraging, where the effectiveness of the LAIV vaccine against all types of laboratory confirmed influenza in children aged 2 to 17 was reported to be 65.8% (95% CI: 30.3–83.2).^14^

This study aimed to evaluate the uptake, direct and indirect impact of the childhood LAIV programme in England in the 2016/2017 influenza season using established influenza surveillance schemes.

## 2 METHODS

Most of the pilot areas which started vaccinating children of primary school age in the 2013/2014 season continued with the programme up to and including the 2016/2017 season.^6^, ^7^, ^8^ The pilot areas in 2016/2017 were the same as those who delivered the programme in the 2015/2016 season. All the pilot areas now deliver the programme through a school‐based delivery method.^8^

### 2.1 Measuring vaccine uptake

**FIGURE 1:**
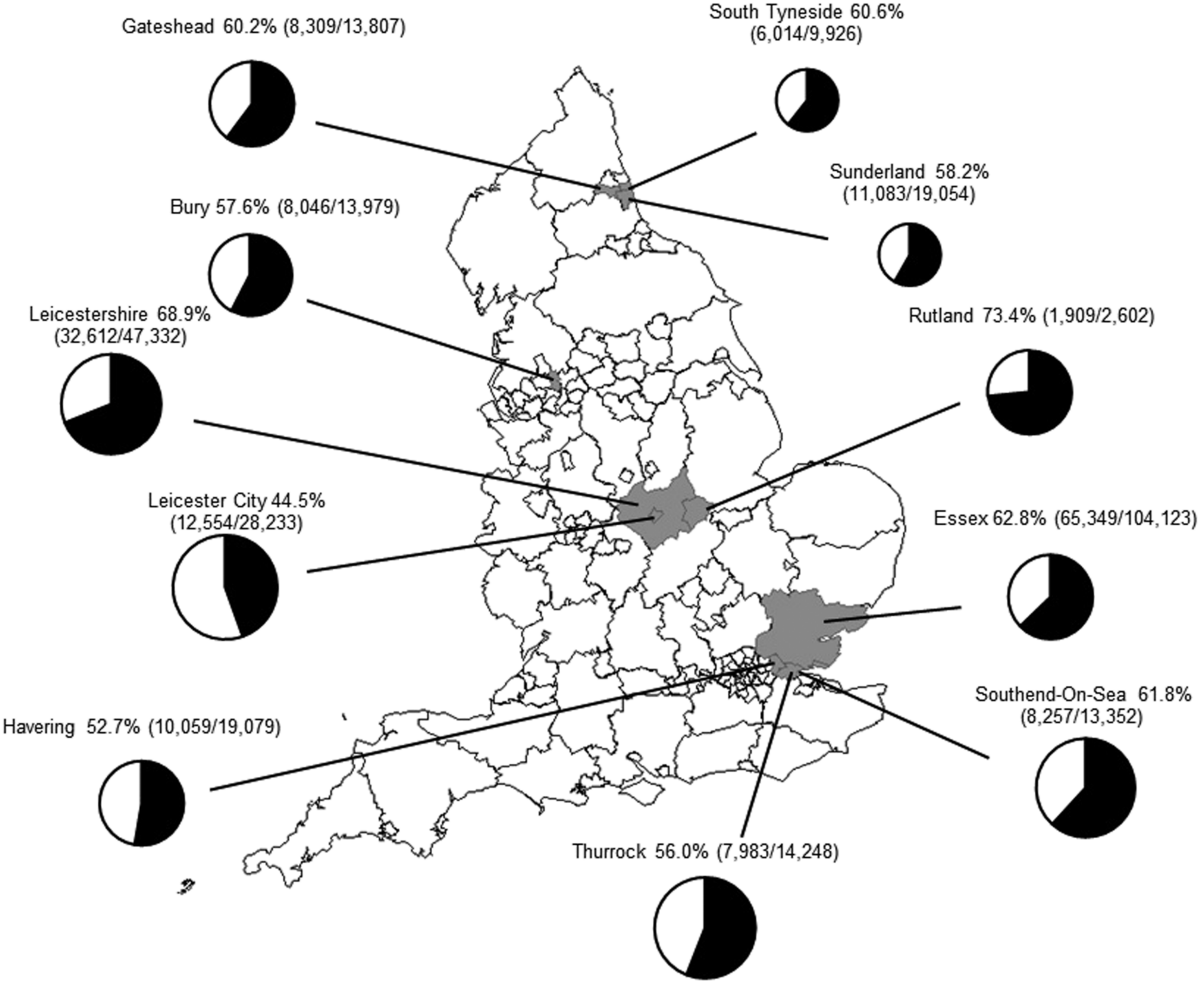
Cumulative uptake of live attenuated influenza vaccine in primacy school age children* in pilot areas, England, 1 September 2016 to 31 January 2017

In the 2016/2017 pilot programme, the children recommended to receive vaccination were defined as those born between 1 September 2005 and 31 August 2011 (aged 5 to 11 years) who resided in five geographically discrete pilot areas in England: Greater Manchester (Bury), Leicestershire and Lincolnshire (Leicester, Leicestershire and Rutland), London (Havering), Essex (Southend‐On‐Sea and Thurrock) and Northumberland Tyne & Wear (Gateshead, South Tyneside and Sunderland), as shown in Figure 1.

An established and standardised web‐based portal, Immform, was used to collate and report vaccine administration data by locally commissioned data providers, to Public Health England (PHE).^15^

The end of season vaccination uptake was calculated as the number of children in the target population who received at least one dose of influenza vaccine in the period from 1 September 2016 until 31 January 2017. Healthy children and those children identified to be at‐risk with no contraindication against the LAIV vaccine were offered the quadrivalent LAIV vaccine. Children identified to be at‐risk in whom LAIV was contraindicated, were offered the quadrivalent inactivated vaccine instead.

### 2.2 Measuring impact

The 2016/2017 LAIV impact study ran from weeks 402 016 to 142 017, by which time influenza transmission in the community had returned to well below baseline levels.^13^

To denote the overall impact of the childhood LAIV programme, cumulative disease incidence in non‐pilot areas was compared against the cumulative disease incidence in pilot areas for a number of virological and clinical respiratory end points in primary and secondary care surveillance schemes, during the period of the study. In addition, the impact of the LAIV programme on excess all‐cause mortality was also studied.

As the dominant circulating influenza A subtype during the study period 2016/2017 was influenza A(H3N2) which is known to affect the older age groups, the previously described 17+ age group was split into two further age groups.^8^ Thus, the overall impact of vaccinating primary school age children was evaluated across five age groups: 5–10 years, to measure the direct impact and <5, 11–16, 17–64 and 65+ years to measure the indirect impact for the 2016/2017 season.

### 2.3 Data sources

Various established influenza surveillance schemes were used to measure the impact of the LAIV programme in the 2016/2017 season, as described below and summarised in Table 1.^13^

**TABLE 1:** Summary of data sources with their respective impact measures and number of non‐pilot and pilot sites

Disease indicators	Data source/scheme	Impact measure	Number of non‐pilot sites^a^	Number of pilot sites^a^
Primary care	GP consultations for ILI	Cumulative ILI consultation rates per 100 000 GP registered population	162	11
	GP swabbing scheme	Cumulative influenza swab positivity (%)	88	4
Secondary care	USISS sentinel scheme	Cumulative hospitalisation rate per 100 000 trust catchment population	20	3
	USISS mandatory scheme	Cumulative ICU/HDU rate per 100 000 trust catchment population	137	11
	RDMS	Cumulative influenza swab positivity (%)	‐	‐
	ED attendances	Cumulative proportion (%) of ED attendances	25	1
Excess all‐cause mortality	All‐cause mortality	Cumulative all‐cause excess mortality rate per 100 000 population	‐	‐
	Respiratory mortality	Cumulative respiratory mortality rate per 100 000 population	‐	‐

*Note*: Hyphen (‐) means non‐pilot/pilot area assignment for these schemes was determined based on the patient/deceased's usual residence postcode.

Abbreviations: ED, emergency department; GP, general practice; ILI, influenza‐like Illness; RDMS, Respiratory DataMart system; USISS, UK Severe Influenza Surveillance System.

^a^ Sites are defined as GP practices for primary care indicators and NHS Trusts/Emergency departments for secondary care indicators.

### 2.4 Primary care

Surveillance in primary care was carried out through quantifying weekly general practice (GP) consultations for influenza‐like illness (ILI) and acute bronchitis in children less than 5 years old through GP practices that are part of the Royal College of General Practitioners (RCGP) Research and Surveillance Centre (RSC) Weekly Returns Service sentinel GP network.^16^ Through this network, 162 GP practices participated in non‐pilot areas and 11 practices in pilot areas.

Respiratory swabbing in primary care is undertaken through GP practices within the RCGP RSC network as well as an additional sentinel swabbing network, the Specialist Microbiology Network (SMN).^17^ Through both schemes, a total of 88 GP swabbing practices participated in non‐pilot areas, and four GP swabbing practices participated in pilot areas. In all areas, swabs were taken from patients presenting with ILI, regardless of the patient's vaccination status.

### 2.5 Secondary care

Surveillance in secondary care was undertaken through four surveillance schemes.

The UK Severe Influenza Surveillance System (USISS) sentinel scheme comprised a network of 23 National Health Service (NHS) hospital trusts across England (20 in non‐pilot areas and three in pilot areas) in the 2016/2017 season. The USISS mandatory scheme comprised all intensive care (ICU) and high dependency units (HDU) in England (137 in non‐pilot areas and 11 in pilot areas) for the 2016/2017 season.^18^

The weekly number of laboratory confirmed influenza hospital admissions and ICU/HDU admissions, collated through the two USISS (sentinel and mandatory) schemes were used to calculate confirmed influenza hospital and ICU/HDU admission rates by the specified age groups and pilot and non‐pilot areas, using estimated hospital catchment population per 100 000 population as the denominator.^19^

The Emergency Department Syndromic Surveillance System (EDSSS), as previously described,^20^ monitored the proportion of all weekly respiratory related emergency department (ED) attendances against all ED attendances with a diagnosis by the specified age group and by pilot (one ED) and non‐pilot (25 EDs) areas.

Respiratory swabbing in secondary care is monitored through the Respiratory DataMart system (RDMS), as over 90% of samples from this scheme are collated from patients in secondary care settings.^21^ Overall swab positivity for all influenza reverse‐transcription‐polymerase chain reaction (RT‐PCR) respiratory swab results in a network of 14 PHE and NHS laboratories in England was compared by age group and by pilot area. Each patient's postcode of residence was used to assign patient samples to a pilot or non‐pilot area.

### 2.6 Excess all‐cause mortality

Excess all‐age all‐cause mortality and all‐age respiratory deaths (defined as ICD‐10J codes) were estimated as the observed weekly number of deaths (corrected for delays in reporting) compared to the expected number of deaths based on historical trends to denote significant excess mortality in both pilot and non‐pilot areas. This was conducted using the European Monitoring of Excess Mortality for Public Health Action (EuroMOMO) standard algorithm^22^ to calculate weekly excess mortality by computing death registration data from the Office for National Statistics (ONS), based on the usual place of residence. The EuroMOMO algorithm was applied to both all‐cause mortality and death registrations where respiratory (ICD 10 “J” code) was the primary cause of death.^23^

### 2.7 Statistical methods

The methods used to evaluate and measure the impact of the LAIV vaccine on the various surveillance schemes were the same as those used in previous seasons.^7^, ^8^

Surveillance schemes where the cumulative disease incidence rates were observed, including the RCGP RSC, USISS sentinel and mandatory schemes, the number of weekly disease incidences between weeks 402 016 to 142 017 were summed over the average weekly population at risk, per 100 000 population. Separate calculations were performed for overall and age group specific rates in non‐pilot and pilot areas, where exact Poisson confidence intervals (CI) were calculated.

For the primary care GP swabbing and the RDMS, cumulative influenza swab positivity was calculated by summing the number of positive samples over the total number of samples tested between weeks 402 016 and 142 017, by age group and pilot area.

For the syndromic surveillance ED scheme, cumulative proportions of respiratory ED attendances were calculated by summing the total number of respiratory coded ED attendances over the total number of ED attendances with a diagnosis between weeks 402 017 and 142 017, by age group and pilot area.

To measure the impact of the LAIV programme, the non‐pilot areas were set as reference, and odds ratios and 95% CI were calculated by age‐group and surveillance scheme. For each scheme, adjusting for clustering at the reporting unit level (e.g., GP practice, hospital or laboratory), data were converted to binomial individual level and random effects logistic regression undertaken.

For excess mortality monitoring, cumulative excess mortality rates were calculated by summing the difference between observed and expected weekly deaths over the time of the study by pilot area and using the resident population respective to non‐pilot or pilot areas.

### 2.8 Laboratory methods

Real‐time PCR methods were used to detect circulating influenza A, B and other respiratory viruses for influenza laboratory confirmation of samples from the various primary and secondary care schemes.^24^ Samples from the RCGP sentinel GP scheme were sent to the PHE Reference Virus Unit, Colindale; samples from the SMN sentinel GP scheme, USISS and Respiratory DataMart schemes were sent to one of the network of specialist PHE microbiology laboratories or NHS laboratories elsewhere in England.

## 3 RESULTS

### 3.1 Vaccine uptake

An estimated 172 175 primary school children aged 5 to 11 years of age in five pilot areas received at least one dose of influenza vaccine during the vaccination campaign period (1 September 2016 to 31 January 2017), with the estimated total target population being 285 735 primary school aged children. This resulted in an overall vaccine uptake of 60.3%.^25^

The uptake ranged from 44.5% to 73.4% by pilot site (Figure 1). Uptake by school year group ranged from 63.0% in Year 1 group (5–6 years) to 57.2% in Year 6 group (10–11 years).

In 2 to 4 year olds, the influenza vaccine uptake delivered through GPs was 44.4% (569 GP practices) in primary school pilot areas compared to 37.6% (6867 GP practices) in non‐pilot areas.

In children of school year groups 1–3, the influenza vaccine uptake was 62.0% (90 322/145 664 children) in primary school pilot areas compared to 54.9% (1 032 905/1 880 516 children) in non‐pilot areas.

### 3.2 Vaccine programme impact

From weeks 402 016 to 142 017, cumulative GP ILI consultation rates and swab positivity in primary and secondary (RDMS) care were lower in pilot areas compared with rates in non‐pilot areas across all age groups.

**FIGURE 2:**
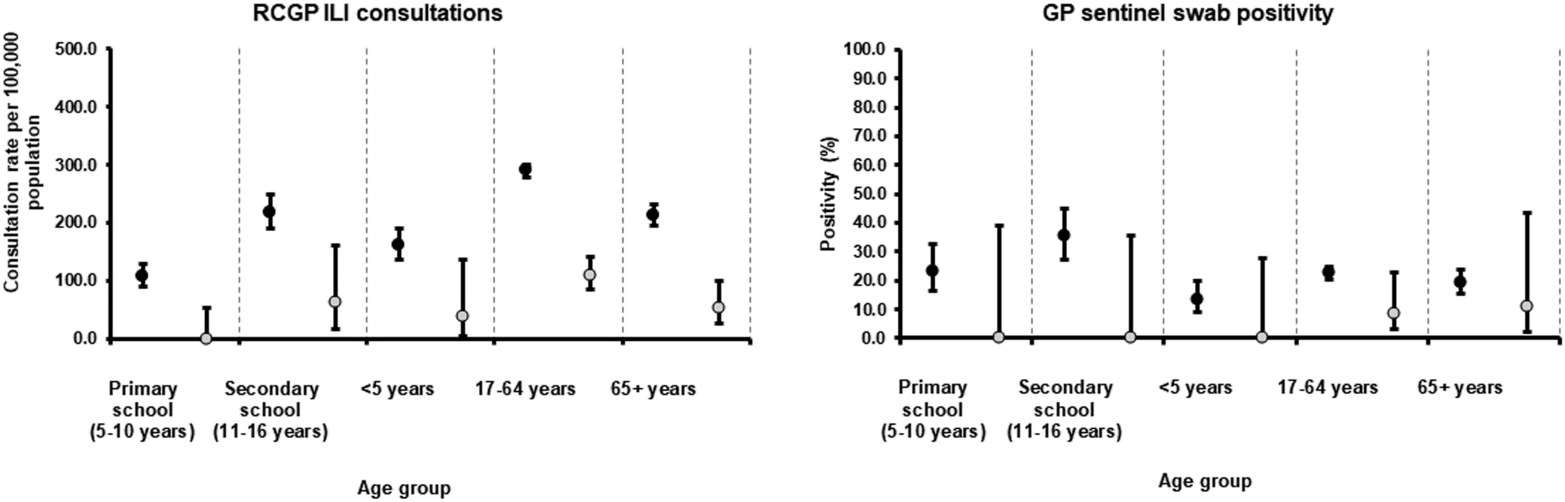
Cumulative primary care schemes in primary school pilot and non‐pilot areas, England, weeks 402 016 to 142 017 with 95% CI (grey = pilot; black = non‐pilot). CI, confidence interval; ILI, influenza‐like illness; RCGP, Royal College of General Practitioners

**FIGURE 3:**
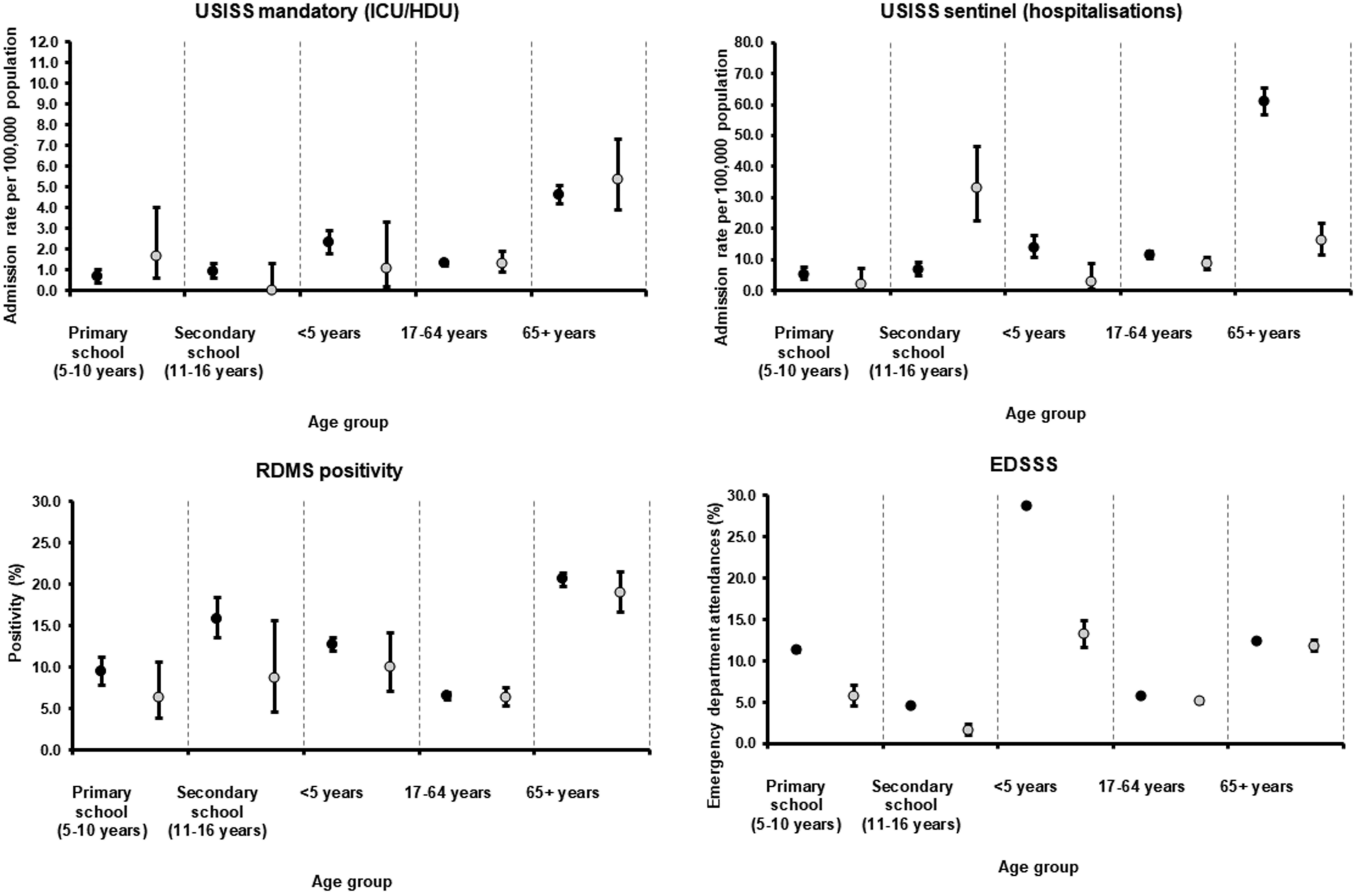
Cumulative secondary care schemes in primary school pilot and non‐pilot areas, England, weeks 402 016 to 142 017 with 95% CI (grey = pilot; black = non‐pilot). CI, confidence interval; EDSSS, Emergency Department Syndromic Surveillance System; USISS, Severe Influenza Sentinel Surveillance System

Such differences were however less marked in secondary care schemes in particular ED respiratory attendances (EDSSS) and ICU/HDU flu confirmed rates (USISS) in the older age groups (17–64 and 65+ years) (Figures 2 and 3).

**FIGURE 4:**
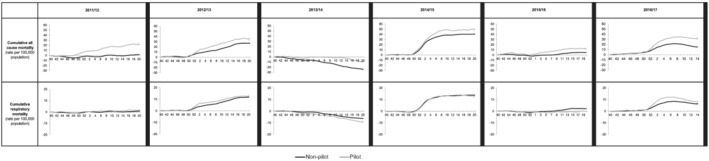
Cumulative weekly all‐cause and respiratory excess mortality rate in primary school pilot and non‐pilot areas, England, influenza season in all ages, weeks 402 011 to 142 017 (grey = pilot; black = non‐pilot)

Cumulative all‐age all‐cause excess mortality by season was significantly higher in pilot areas both pre‐introduction (2011/2012 and 2012/2013) and post‐introduction (2013/2014 to 2016/2017) of the childhood vaccination programme, as previously observed. By way of contrast, cumulative all‐age respiratory related excess mortality was significantly higher in pilot areas in the seasons pre‐introduction of the programme; however, in the post‐introduction period of the programme, excess mortality was significantly lower in 2013/2014 and 2015/2016 (Figure 4).

**FIGURE 5:**
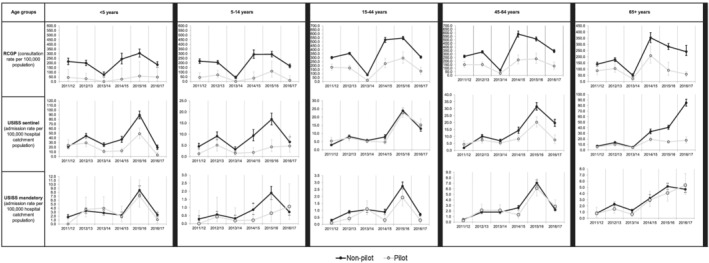
Cumulative, age‐specific influenza surveillance schemes in pilot and non‐pilot areas before (2011/2012 and 2012/2013) and after (2012/2013; 2014/2015; 2015/2016; 2016/2017) vaccine programme introduction, England (grey = pilot; black = non‐pilot). ILI, influenza‐like illness; RCGP, Royal College of General Practitioners; USISS, Severe Influenza Sentinel Surveillance System. *For the 2013–2014, the data are based on weeks 40 to 152 014 as per the previously published data. The pilot areas which were established for the 2013–2014 season were used to calculate the rates by age groups. *For the 2014–2015, the data are based on weeks 40 to 142 015 as per the previously published data. The pilot areas which were established for the 2014–2015 season were used to calculate the rates by age groups; this excludes secondary school pilots. *For the 2011–2012, 2012/2013, 2015/2016 and 2016/2017 seasons, the 2016/2017 pilot areas were applied to the data

Examination of pre‐vaccination data for those schemes for which data were available (RCGP RSC ILI and influenza confirmed hospitalisations and ICU admissions), provided a mixed pattern. Cumulative ICU/HDU admission and hospitalisation rates for influenza were similar in primary school pilot compared to non‐pilot areas for the two seasons prior to the start of the childhood vaccination programme, whereas for the three seasons since, they have been consistently lower. The differences were less marked for GP ILI consultation rates, where pre‐introduction ILI rates were generally lower in pilot compared to non‐pilot areas, which may reflect underlying differences between the pilot and non‐pilot areas (Figure 5).

### 3.3 Adjusted impact for primary and secondary care schemes

For primary care schemes, significant reductions were observed in cumulative GP ILI consultation rates in adults in pilot areas compared to non‐pilot areas when adjusting for clustering, as a result of vaccinating primary school aged children (Table 2). Non‐significant reductions were also noted in children of primary school age and in under 5 year old children for GP ILI consultation rates in pilot compared to non‐pilot. Non‐significant reductions were also observed in cumulative swab positivity in both adults and children (Table 2).

**TABLE 2:** Adjusted impact of vaccinating primary school age children on selected primary care influenza surveillance schemes, England, influenza season, weeks 402 016 to 142 017

Age group		Measure	RCGP (per 100 000 population)		Sentinel swab positivity (%)	
			Non‐pilot	Pilot	Non‐pilot	Pilot
Primary school	5–10 years	rate	107.9	0.0	23.5	0
		(*n*/*N*)	(120/111 185)	(0/6793)	(23/98)	(0/6)
		Risk difference		−107.9		−23.5
		Odds ratio		0.00 (0.00 to 0.52)		0.00 (0.00 to 2.19)
		*p* value		0.001*		0.334
Secondary school	11–16 years	rate	218.3	62.4	35.5	0.0
		(*n*/*N*)	(220/100 780)	(4/6415)	(39/110)	(0/7)
		Risk difference		−155.9		−35.5
		Odds ratio		0.31 (0.09 to 1.08)		0.00 (0.00 to 1.03)
		*p* value		0.066		0.093
Other age groups	<5 years	rate	161.4	37.8	13.5	0.0
		(*n*/*N*)	(144/89 234)	(2/5286)	(21/156)	(0/10)
		Risk difference		−123.6		−13.5
		Odds ratio		0.36 (0.05 to 2.49)		0.00 (0.00 to 2.56)
		*p* value		0.300		0.365
	17–64 years	rate	289.9	110.5	22.6	8.8
		(*n*/*N*)	(2941/1 014 483)	(63/57 021)	(318/1407)	(3/34)
		Risk difference		−179.4		−13.8
		Odds ratio		0.40 (0.20 to 0.78)		0.35 (0.09 to 1.35)
		*p* value		0.007*		0.127
	65+ years	rate	212.7	53.8	19.2	11.1
		(*n*/*N*)	(575/270 312)	(10/18 584)	(71/369)	(1/9)
		Risk difference		−158.9		−8.1
		Odds ratio		0.31 (0.12 to 0.81)		0.52 (0.05 to 5.25)
		*p* value		0.017*		0.579

Abbreviations: CI, confidence interval; RCGP, Royal College of General Practitioners.

* *p* values <0.05.

For the secondary care schemes, non‐significant reductions were seen in all but one indicator (USISS mandatory—ICU admissions) in 5 to 10 year old children in pilot areas compared to non‐pilot areas (Table 3). Non‐significant reductions were also noted in all but one (11 to 16 year olds) age groups for influenza confirmed hospitalisations (USISS sentinel) and RDMS positivity (Table 3). A significantly higher rate for influenza confirmed hospitalisations in pilot areas for 11 to 16 year olds were noted through the USISS sentinel scheme (Table 3).

**TABLE 3:** Adjusted impact of vaccinating primary and/or secondary school age children on selected secondary care influenza surveillance schemes, England, influenza season, weeks 402 016 to 142 017

Age group		Measure	USISS mandatory (per 100 000 population)		USISS sentinel (per 100 000 population)		Respiratory DataMart system (RDMS) (%)		Emergency department respiratory attendances (%)	
			Non‐pilot	Pilot	Non‐pilot	Pilot	Non‐pilot	Pilot	Non‐pilot	Pilot
Primary school	5–10 years	rate	0.7	1.7	5.2	1.9	9.4	6.4	11.4	5.7
		(*n*/*N*)	(22/3377936)	(5/291687)	(29/552768)	(2/102732)	(115/1219)	(13/203)	(6118/53806)	(75/1308)
		Risk difference		1.0		−3.3		−3.0		−5.7
		Odds ratio		1.75 (0.27 to 11.52)		0.49 (0.07 to 3.43)		0.75 (0.35 to 1.64)		0.71 (0.15 to 3.34)
		*p* value		0.561		0.477		0.478		0.666
Secondary school	11–16 years	rate	0.9	0.0	6.7	33.0	15.8	8.7	4.6	1.6
		(*n*/*N*)	(31/3 282 428)	(0/281 903)	(35/522 467)	(32/97 105)	(136/859)	(9/104)	(2639/57 882)	(30/1859)
		Risk difference		−0.9		26.3		−7.1		−3.0
		Odds ratio		0.00 (0.00 to 1.44)		11.5 (1.83 to 71.9)		0.68 (0.29 to 1.57)		0.47 (0.14 to 1.61)
		*p* value		0.172		0.009*		0.361		0.233
Other age groups	<5 years	rate	2.3	1.1	13.9	3.0	12.7	10.1	28.7	13.2
		(*n*/*N*)	(69/3 031 148)	(3/269 118)	(69/487 979)	(3/101 577)	(1002/7881)	(28/278)	(30 938/107 690)	(217/1642)
		Risk difference		−1.2		−10.9		−2.6		−15.5
		Odds ratio		0.64 (0.09 to 4.58)		0.40 (0.04 to 4.05)		1.04 (0.60 to 1.80)		0.55 (0.11 to 2.81)
		*p* value		0.660		0.436		0.903		0.469
	17–64 years	rate	1.3	1.4	11.6	8.7	6.5	6.4	5.7	5.1
		(*n*/*N*)	(380/29 677 462)	(36/2 668 799)	(494/4 314 066)	(80/923 457)	(912/13 995)	(134/2107)	(31 682/554 510)	(956/18 880)
		Risk difference		0.1		−2.9		−0.1		−0.6
		Odds ratio		0.96 (0.50 to 1.85)		0.98 (0.19 to 5.08)		0.88 (0.69 to 1.11)		1.13 (0.30 to 4.35)
		p value		0.900		0.981		0.281		0.855
	65+ years	rate	4.6	5.4	60.9	16.1	20.6	19.0	11.8	12.4
		(*n*/*N*)	(380/8 190 916)	(41/763 284)	(751/1 232 675)	(40/248 656)	(2473/12 028)	(192/1013)	(24 839/199 721)	(1099/9276)
		Risk difference		0.7		−44.8		−1.6		0.6
		Odds ratio		0.88 (0.43 to 1.85)		0.26 (0.02 to 3.95)		0.94 (0.77 to 1.14)		1.30 (0.28 to 6.09)
		p value		0.753		0.335		0.511		0.736

Abbreviations: CI, confidence interval; RDMS, Respiratory DataMart system; USISS, UK Severe Influenza Surveillance System.

* *p* values <0.05.

## 4 CONCLUSION

This study evaluates the uptake and impact of the childhood LAIV influenza vaccine programme in its fourth season of its phased introduction in England. The programme continued to target children of primary school age in discrete geographical pilot areas in the 2016/2017 season, in addition to its national implementation. As in previous seasons, higher or similar levels of vaccine uptake amongst children of primary school age continue to be reported in the targeted pilot population. We also demonstrate that populations with vaccinated children of primary school age are associated with reductions, although not always significant, in the incidence of influenza for a range of surveillance schemes in comparisons to populations without vaccinated children of primary school age. We also observed higher all‐cause and respiratory excess deaths in pilot compared to non‐pilot areas—both before and after introduction of LAIV.

Also, as in previous seasons, good vaccine uptake levels were observed with the targeted population achieving 7.1% higher uptake than the non‐targeted population. Amongst the targeted population, almost 55% of pilot areas achieved a vaccine uptake in excess of 60%. This reinforces the choice of a school based administration to achieve high childhood vaccination uptake.^26^ The 2016/2017 season saw the circulation of influenza A(H3N2) predominantly followed with a small number of influenza B detections in the latter part of the season, affecting the older population as expected for this virus. Despite this, significant indirect reductions were observed in the older age groups particularly amongst primary care indicators. Non‐significant reductions were noted amongst primary school aged children for primary care consultations and swab positivity, secondary care swab positivity, laboratory confirmed hospitalisations and ED attendances. As shown previously and in other studies, it continues to be evident that the impact and greater effect sizes are in primary care indicators in comparison to those in secondary care, in both targeted and non‐targeted populations.^26^, ^27^, ^28^, ^29^

Findings from historical trends for GP ILI consultations, hospitalisations and ICU/admissions suggest significant differences post‐programme introduction in GP ILI consultations in all age groups; however, these differences should be interpreted with caution due to pre‐existing differences between the pilot and non‐pilot areas prior to the programme. Nonetheless, as seen previously, reductions remain evident in targeted populations and there continues to be evidence that an indirect effect on the <5 year olds is present. This is also highlighted in a recent study by Benjamin‐Chung et al.,^26^ where higher influenza vaccine uptake as well as greater reductions in influenza‐illness related school absences in targeted and non‐targeted populations were achieved in schools delivering the influenza vaccination programme on site in comparison to schools (matched for similar characteristics) whom did not have a school based delivery programme.

Although there was no evidence of significant adjusted vaccine effectiveness for the >65 year old vaccine programme for the 2016/2017 season,^14^ we still observed significant reductions in GP ILI consultations in this age group in pilot areas compared to non‐pilot areas but not for ICU/HDU admissions. This suggests important possible beneficial indirect effects of the childhood programme on older age groups.

Our finding that excess all‐cause mortality was significantly higher in pilot areas is one that has been noted in previous seasons and can be likely explained by the pre‐existing higher all‐cause excess seen in the pilot areas prior to the introduction of the LAIV programme, which may reflect differences in the underlying health and socio‐demographic profile of the populations in pilot and non‐pilot areas.^7^, ^8^ The higher excess respiratory mortality observed in the pilot areas in our study was surprising as the pattern was reversed in previous post‐programme seasons where higher excess respiratory mortality was noted in non‐pilot areas.^6^, ^8^ Absence of reductions in pneumonia and influenza mortality associated with a school children's vaccination programme has also been reported elsewhere.^30^ This observation may be due to the lack of study power or to the increasingly narrowing gap between the pilot and non‐pilot areas described further below and warrants further investigation.

A number of strengths have been highlighted from this study. First, this study uses data collected from a wide range of well‐established surveillance systems which cover healthcare service utilisation across the disease spectrum of influenza. Second, the methods used in this study to assess the uptake and impact of the childhood programme have been developed over the past three seasons which has enabled us to confidently assess its findings. Third, vaccine uptake is measured at population level which allows for direct comparisons to previous published studies.^6^, ^7^, ^8^

There are some potential limitations to this study, including potential differential reporting. New GP practices and hospitals are recruited each season in pilot and non‐pilot areas which may contribute to higher differential reporting and in turn introduce less sensitive case detection amongst these new entities compared to long‐standing participating practices. There was only one emergency department site in the pilot areas compared to 25 in the non‐pilots areas, although this is taken into account in the model. As the national vaccination programme continues to roll out to children in other school years and the gap between pilot and non‐pilot areas decreases, the ability to carry out such comparisons is diminishing, which is evident when looking at the historical comparisons.

In conclusion, this study is the fourth of its kind in a series of evaluations of the LAIV programme to find continuing positive outcomes in support of the roll out of the national childhood LAIV programme. Other approaches to estimate the population impact of the programme may need to be considered as the narrowing differences between uptake between pilot and non‐pilot areas means that the methods used in this study may not be suitable in future seasons. Lack of impact of the programme on excess all‐cause mortality also warrants further investigation.

## CONFLICT OF INTEREST

The authors declare no conflict of interest.

## AUTHOR CONTRIBUTIONS

**Fiona Warburton:** Conceptualization; data curation; formal analysis; methodology. **Nick Andrews:** Conceptualization; formal analysis; methodology. **nicola boddington:** Data curation. **Hongxin Zhao:** Data curation. **Joanna Ellis:** Data curation. **Elise Tessier:** Data curation. **Matthew Donati:** Data curation. **Alex Elliot:** Data curation. **Helen Hughes:** Data curation. **Rachel Byford:** Data curation. **Gillian Smith:** Data curation. **Manasa Tripathy:** Data curation. **Simon De Lusignan:** Data curation. **Maria Zambon:** Data curation. **Richard Pebody:** Conceptualization; methodology; supervision.

### PEER REVIEW

The peer review history for this article is available at https://publons.com/publon/10.1111/irv.12898.

## Data Availability

Applications for requests to access relevant anonymised data should be submitted to the PHE Office for Data Release (https://www.gov.uk/government/publications/accessing-public-health-england-data/about-the-phe-odr-and-accessing-data/).
