# Correction to ‘Uptake and Impact of Vaccinating Primary School Children Against Influenza: Experiences in the Fourth Season of the Live Attenuated Influenza Vaccination Programme, England, 2016/2017’

**DOI:** 10.1111/irv.13349

**Published:** 2024-07-10

M. A. Sinnathamby , F. Warburton , N. Andrews , N. L. Boddington , H. Zhao , J. Ellis , E. Tessier , M. Donati , A. J. Elliot , H. E. Hughes , R. Byford , G. E. Smith , M. Tripathy , S. de Lusignan , M. Zambon , R. G. Pebody , “Uptake and Impact of Vaccinating Primary School Children Against Influenza: Experiences in the Fourth Season of the Live Attenuated Influenza Vaccination Programme, England, 2016/2017,” Influenza and Other Respiratory Viruses 16, no. 1 (2022): 113–124, 10.1111/irv.12898.34405555 PMC8692804

In the ‘Author Contributions’, the contributions of Mary A. Sinnathamby is missing. It should read as:

## Author Contributions

**Mary Sinnathamby:** conceptualization, data curation, formal analysis, methodology, writing–original draft, writing–review and editing. **Fiona Warburton:** conceptualization, data curation, formal analysis, methodology. **Nick Andrews:** conceptualization, formal analysis, methodology. **Nicola Boddington:** data curation. **Hongxin Zhao:** data curation. **Joanna Ellis:** data curation. **Elise Tessier:** data curation. **Matthew Donati:** data curation. **Alex Elliot:** data curation. **Helen Hughes:** data curation. **Rachel Byford:** data curation. **Gillian Smith:** data curation. **Manasa Tripathy:** data curation. **Simon de Lusignan:** data curation. **Maria Zambon:** data curation. **Richard Pebody:** conceptualization, methodology, supervision.

We apologize for this error.

